# The Development of TIM-Barrel Based Multi-Epitope Protein for *Toxoplasma gondii* Serological Detection in Cats

**DOI:** 10.3390/ani15131893

**Published:** 2025-06-26

**Authors:** Preeyanuch Thongpoo, Jiravich Methawiroon, Bandid Mangkit, Rucksak Rucksaken, Metita Sussadee, Warin Rangubpit, Sasimanas Unajak, Sathaporn Jittapalapong, Eukote Suwan

**Affiliations:** 1 Program of Science and Mathematics (Biology), Faculty of Science and Technology, Phuket Rajabhat University, Phuket 83000, Thailand; preeyanuch@pkru.ac.th; 2 Department of Veterinary Technology, Faculty of Veterinary Technology, Kasetsart University, Bangkok 10900, Thailand; cvtjim@ku.ac.th (J.M.); fvetbdm@ku.ac.th (B.M.); warin.rangubpit@njit.edu (W.R.); fvetspj@ku.ac.th (S.J.); 3 Department of Veterinary Nursing, Faculty of Veterinary Technology, Kasetsart University, Bangkok 10900, Thailand; cvtrsr@ku.ac.th (R.R.); cvtmts@ku.ac.th (M.S.); 4 Kasetsart Vaccines and Bio-Product Innovation Centre (KU-V-BIC), Kasetsart University, Bangkok 10900, Thailand; sasimanas.u@ku.th; 5 Department of Physics, New Jersey Institute of Technology, Newark, NJ 07102, USA; 6 Department of Biochemistry, Faculty of Science, Kasetsart University, Bangkok 10900, Thailand; 7 Center for Advanced Studies in Nanotechnology for Chemical, Food and Agricultural Industries, KU Institute for Advanced Studies, Kasetsart University, Bangkok 10900, Thailand

**Keywords:** *Toxoplasma gondii*, serological methods, indirect ELISA, chimeric multi-epitope protein, TIM-barrel structure

## Abstract

*Toxoplasma gondii*, a pathogen of significant concern in animal production, companion animal health, and public health, particularly affects immunocompromised individuals and pregnant women. Current diagnostic techniques employ both direct and indirect methods, with serological assays widely used for detecting *T. gondii* infections in humans and animals. In this study, the TIM-barrel structure of Br2 *β*-glucosidase was engineered to create 10 chimeric multi-epitope proteins for *T. gondii* serological detection. Indirect ELISA screening identified three promising candidate proteins, V4Z, SFF, and S7V-V4Z-SFF, with sensitivities ranging from 71–86% and specificities ranging from 68–76%. Among these, ELISA-V4Z achieved the highest concordance with the reference IFAT method (Kappa = 0.58, 95% CI = 0.32–0.84) and demonstrated a moderate positive predictive value (PPV, 67%) and strong negative predictive value (NPV, 90%). These results suggest that the V4Z chimeric protein demonstrated the strongest performance among the tested candidates for *T. gondii* detection, exhibiting the highest sensitivity and specificity along with moderate agreement with the reference IFAT. However, its overall diagnostic performance remains limited. These findings highlight the need for further refinement and validation to enhance its diagnostic potential and assess its applicability for broader serological testing.

## Simple Summary

*Toxoplasma gondii* causes toxoplasmosis, which can develop into severe symptoms in pregnant and immunocompromised people. The *T. gondii* diagnosis relies on both direct and indirect methods with various specificities and sensitivities. The serodiagnosis has been introduced to *T. gondii* detection such as latex agglutination test (LAT), indirect immunofluorescent assay (IFAT), and enzyme-linked immunosorbent assays (ELISA). Recombinant antigens are commonly used in serodiagnosis and combinations of recombinant antigens have shown improved serodiagnosis efficacy. In this study, TIM-barrel-based proteins containing *T. gondii* B- and T-cell epitopes were used to detect *T. gondii* infection in cats via ELISA assay. Among 10 chimeric protein constructions, V4Z revealed 86% sensitivity and 76% specificity with moderate agreement with the reference IFAT. These findings suggest that TIM-barrel-based multi-epitope proteins are promising candidates for chimeric antigens in serological detection, which could be applied in pathogen detection by incorporating immunodominant epitopes.

## 1. Introduction

*Toxoplasma gondii* is an obligate intracellular protozoan and one of the most significant zoonotic pathogens, widely distributed among humans and nearly all warm-blooded animals. Its prevalence poses substantial challenges to public health, animal production, and companion animal health globally, particularly due to the role of cats as definitive hosts and the impact on immunosuppressed companion animals [1,2,3]. *T. gondii* transmission occurs primarily through the ingestion of raw or improperly cooked animal products (meat, milk, eggs) containing tissue cysts or through the consumption of contaminated vegetables, fruits, or water with oocysts shed by infected cats, the definitive hosts [4]. In immunocompetent adults, primary *T. gondii* infections are often asymptomatic or manifest as mild, flu-like illnesses. However, in immunosuppressed individuals, acute cerebral or systemic diseases can develop. Pregnant women infected with *T. gondii* may experience miscarriage or stillbirth, and the infection is associated with several neurological disorders in both mothers and newborns. Recent systematic reviews estimate that 25–33% of the global population is seropositive for *T. gondii*, with notable regional variation: Africa (61.4%), Oceania (38.5%), South America (31.2%), Europe (29.6%), North America (17.5%), and Asia (16.4%). In companion animals, seroprevalence of *T. gondii* varies considerably, with reported ranges in cats from 11% to over 70%, depending on region and diagnostic method. [5,6,7,8,9]. In Thailand, over three million cats live in close proximity to humans and significantly contribute to the transmission of *T. gondii* to humans [1,10]. The effective prevention, control, and treatment of toxoplasmosis are hindered by the often asymptomatic or non-specific clinical presentations of the disease, underscoring the need for highly sensitive and specific diagnostic methods [11]. Diagnostic approaches are broadly categorized into direct and indirect methods. Direct methods, including microscopic examination, polymerase chain reaction (PCR), and bioassays, are high specificity but low sensitivity as they require the presence of detectable parasites in the sample. Conversely, indirect methods, particularly serological assays such as the dye test (DT), direct or modified agglutination test (DAT/MAT), indirect hemagglutination test (IHA), latex agglutination test (LAT), indirect immunofluorescent assay (IFAT), and enzyme-linked immunosorbent assays (ELISA), are currently preferred for routine *T. gondii* diagnosis in both humans and animals due to their practicality and cost-effectiveness [12,13,14]. Among these serological tests, ELISA stands out for its reliability, practicality, and economical advantages, making it widely adopted for detecting *T. gondii* infections in animals. While commercial ELISA kits are available, there is also a significant emphasis on developing in-house ELISAs utilizing novel antigens and antibodies to enhance diagnostic performance [15,16].

Three main types of antigens, including native, recombinant, and chimeric proteins, have been used for *T. gondii* diagnosis. Native antigens, typically derived from whole tachyzoites or tachyzoite-based products, require culturing, loss during purification, batch-to-batch variability, and complicating standardization. Recombinant proteins are a viable alternative for producing antigens for serological diagnosis [13,14,17]. Recombinant antigens include dense granule proteins (GRA), cyst matrix antigens (MAG), microneme proteins (MIC), surface antigens (SAG), rhoptry antigens (ROP), and various peptide fragments [12,13,14,16]. Among these, GRAs, particularly GRA7, are extensively studied and have demonstrated sensitivities and specificities ranging from 35.1 to 89.7% and 89.9 to 96.0%, respectively, in feline studies [17,18,19]. Moreover, combinations of recombinant antigens (e.g., SAG2 + GRA2 + GRA6 + GRA7 + GRA15) have shown improved diagnostic performance, achieving sensitivities of 89.2% and specificities of 95.4% compared to single-antigen assays [18]. Chimeric antigens represent a significant advancement in diagnostic approach by incorporating multiple epitopes to enhance sensitivity and specificity. [13,16,20]. Computational methods facilitate the prediction of immunoreactive epitopes based on the physicochemical properties of amino acids, enabling the design of antigens recognized by T-cell and B-cell receptors [21,22,23,24,25]. This approach has been used in various pathogens with high accuracy and no cross-reactivity in Mayaro virus [26], 98.8% sensitivity and 100% specificity in West Nile virus [27], and 100% sensitivity and specificity in *Trypanosoma cruzi* [28] detections. However, only a few studies have investigated the use of chimeric antigens for detecting *T. gondii* in animals such as horses, pigs, sheep, and goats, with reported sensitivities ranging from 28.4% to 100% and specificities from 95.1% to 100% [29,30,31]. Notably, only a SAG1-GRA8 chimeric antigen has been used to detect *T. gondii* in cats [32]. In addition, most of the current studies on *T. gondii* epitopes have focused on characterized proteins, which may lose some potential proteins, especially uncharacterized proteins [33]. A classical (*β*/*α*)_8_-TIM barrel structure contains the central eight *β*-sheet surrounded by the eight *α*-helices. The *β*-strand core interconnects with *βα*-loops to form a catalytic face and *αβ*-loops to form a stable face of protein structure. The outer *α*-helical surfaces interact with other proteins, peptides, or substrates, which may show potential for multi-epitope replacement and be used in serological diagnosis [34].

This study aims to engineer a Br2 *β*-glucosidase, derived from the bovine rumen metagenome [35] and characterized by a classical (*β*/*α*)_8_-TIM barrel structure [36]. The outer *α*-helices were replaced by a novel group of B-cell and T-cell epitopes, identified from *T. gondii* membrane proteins through a genome-wide screening by Li et al. [33], to construct the multi-epitope proteins for serological detection of *T. gondii* infections in cat.

## 2. Materials and Methods

### 2.1. Cat Serum Preparation and ETHICAL Approval

Serum samples from 39 mixed-breed cats (17 females and 22 males), aged 1 to 7 years (mean ± standard deviation: 2.13 ± 1.34 years), were obtained from a previous study [19]. These animals were sampled from the Rabies Control Division, Buddhist temples, and animal clinics within the Bangkok metropolitan area, Thailand. Blood samples (1–2 mL) were kept in sterile plain tubes, centrifuged to separate serum, and stored at −70 °C until used. Ethical approval for animal use in this research was obtained from the Kasetsart University Institutional Animal Care and Use Committee (Approval No.: ACKU-60-VTN-007), Kasetsart University, Bangkok, Thailand.

### 2.2. Constructions of Chimeric Multi-Epitopes

Chimeric multi-epitope constructs were engineered by modifying the recombinant plasmid pET15b-*Br2* [35] using the Q5^®^ Site-Directed Mutagenesis Kit (New England Biolabs, Inc., Massachusetts, USA) according to the manufacturer’s instructions. Large-scale screening of *T. gondii* membrane proteins from the UniProt database [37] was performed to identify B-cell and T-cell epitopes by Li, et al. [33]. Based on sequence similarities, S7VVK4 (S7V), V4ZJG5 (V4Z), A0A125YIN1 (A125Y), S8F3F6-1 (SFF), and S8EYA7 (S8E) were selected to replace outer *α*-helices of TIM-barrel structure. The helix 3 (H3) was replaced with S7V, while helix 4 (H4) was replaced with A125Y or SFF, and helix 6 (H6) was replaced with S8E or V4Z. Primer pairs were designed using NEBaseChanger software to facilitate precise epitope insertions (Table S1).

The construction process involved a two-step PCR protocol. Initially, the target helix was deleted from the plasmid pET15b-*Br2*, followed by simultaneous phosphorylation, ligation, and template removal using a Kinase, Ligase, and *Dpn*I (KLD) enzyme mixture, which yielded a deleted plasmid. Subsequently, specific primers were used to amplify and insert the selected epitopes into the deleted plasmid, followed by KLD reactions. The resulting plasmids were then transformed into *E. coli* NEB5*α* competent cells. Positive clones underwent plasmid extraction, DNA sequencing (ATGC Co., Ltd., Pathum Thani, Thailand), and sequence alignment using Clustal Omega (https://www.ebi.ac.uk/jdispatcher/msa/clustalo (accessed on 19 November 2023)) [38] and Jalview software version 2 [39] to confirm successful mutations and insertion accuracy. The confirmed constructs were further modified to produce multi-epitope combinations, resulting in a set of ten chimeric constructs, which included single, double, and triple epitope insertions.

### 2.3. Productions of Chimeric Multi-Epitope Proteins

The confirmed chimeric plasmids were transformed into *E. coli* BL21(pLysE) cells for expression. A single colony of each chimeric clone was inoculated into Luria–Bertani (LB) broth and grown overnight at 37 °C with shaking at 220 rpm. The culture was diluted 1:100 into fresh LB broth and grown until the OD_600nm_ reached 0.4–0.6. The protein expression was induced with 0.1 mM isopropyl *β*-D-1-thiogalactopyranoside (IPTG) and incubated for an additional 3 h. Cells were harvested, lysed by sonication, and centrifuged to collect the supernatant containing the expressed chimeric protein. Proteins were purified using Ni^2+^-NTA affinity chromatography (GE Healthcare, Uppsala, Sweden) and eluted in imidazole buffer. Purity and concentration of the purified proteins were verified by 10% SDS-PAGE and Bradford assay.

### 2.4. Indirect ELISA Condition and Candidate Chimeric Multi-Epitopes Testing

Indirect ELISA was performed to identify the optimal concentration and efficacy of each chimeric multi-epitope protein for detecting *T. gondii* infection in cat sera. Microplate wells were coated with 0.5, 0.75, or 1.5 µg/mL of each protein at 37 °C for 2 h. Plates were blocked with 7% skim milk in phosphate-buffered saline (PBS, pH 7.4) for 1 h at 37 °C. Diluted cat sera (1:100) from three true positive and three true negative samples [18] were added and incubated at 25 °C with shaking. The plates were washed with PBS containing 0.1% Tween 20, then incubated with a 1:8000 dilution of peroxidase-conjugated AffiniPure Goat Anti-Cat IgG (H + L) (Jackson ImmunoResearch Laboratories Inc., West Grove, PA, USA) for 30 min at 25 °C. After further washing, TMB substrate (Avantor, Inc., Radnor, PA, USA) was added and incubated in the dark for 30 min. Absorbance at 655 nm was measured using an iMark™ Microplate Absorbance Reader with Microplate Manager^®^ 6 software (Bio-Rad Laboratories, Inc., Hercules, CA, USA) to determine the optimal concentration and distinguish between positive and negative samples.

### 2.5. Evaluation of Chimeric-Multi-Epitopes and Statistical Analysis

To evaluate the diagnostic efficacy of selected chimeric multi-epitope proteins, ELISA was performed on a panel of 39 cat sera previously categorized by Suwan, et al. [19] as either positive or negative for *T. gondii*. The panel included 13 confirmed positive samples, 5 false positives, 20 confirmed negatives, and 1 false negative. The ELISA protocol described above was used, with a protein concentration of 0.5 µg/mL. The statistical analyses were conducted using version 4.4.1 of the R programming language [40]. A 95% confidence interval (CI) was applied, and *p*-Value < 0.05 was considered significant. To indicate the precision and reliability of the assay, the intra-assay coefficient of variation (CV) was calculated. The ELISA results were analyzed using the ‘epiR’ package in R to determine the sensitivity, specificity, positive predictive value (PPV), and negative predictive value (NPV) of each candidate chimeric protein, by comparing with the IFAT results serving as the reference standard. The diagnostic performance of each ELISA assay across various cutoff points was evaluated using receiver operating characteristic (ROC) curve analysis with the ‘ROCR’ package in the R program. The accuracy of area under the curve (AUC) values was determined using the same package and evaluated following the guidelines established by Swets [41] and Reynoso-Palomar et al. [42]. AUC values were interpreted as follows: non-informative (AUC < 0.5), low accuracy (0.5 ≤ AUC < 0.7), moderate accuracy (0.7 ≤ AUC < 0.9), and high accuracy (0.9 ≤ AUC ≤ 1.0). Additionally, McNemar’s test, implemented using the ‘mcnemar.test’ function in R, was utilized to compare the prevalence between the assays [43]. Moreover, the agreement between ELISA and IFAT results was evaluated using Cohen’s Kappa coefficient with the ‘fmsb’ package in R, and the Kappa values were interpreted according to the guidelines established by Landis and Koch [44]. Furthermore, to evaluate the agreement between ELISA and IFAT results while accounting for prevalence and bias, the prevalence-adjusted bias-adjusted Kappa (PABAK) was evaluated using the ‘epi.kappa’ function, thereby enhancing the accuracy of agreement estimates.

## 3. Results

### 3.1. Chimeric Multi-Epitopes

**Figure 1:**
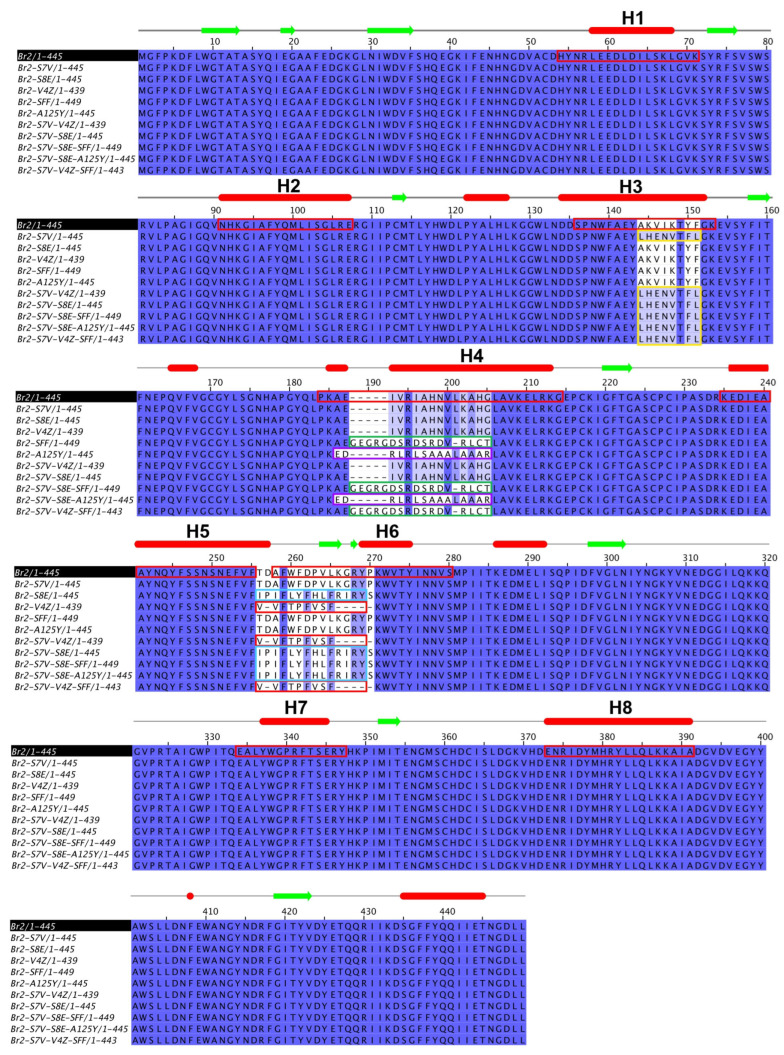
The scheme of multiple sequence alignment between Br2 wildtype and 10 chimeric multi-epitope proteins. The secondary structure was shown the prediction for wild type (*α*-helices in red bars and *β*-sheet in green arrows). The *α*-helix residues of Br2 were shown in red boxes. The inserted residues of S7V, SFF, A125Y, V4Z, and S8E were shown in yellow, green, magenta, pink, and cyan boxes, respectively.

Ten chimeric multi-epitope proteins were successfully constructed, encompassing various epitope configurations, including single (A125Y, S7V, S8E, SFF, and V4Z), double (S7V-V4Z, S7V-S8E), and triple insertions (S7V-V4Z-SFF, S7V-S8E-SFF, and S7V-S8E-A125Y). Each protein was engineered by substituting specific *α*-helix regions of Br2 *β*-glucosidase with selected *T. gondii* B-cell and T-cell epitopes (Figure 1). Specifically, helix 3 of Br2 was replaced with S7V (a guanylate cyclase, predicted T-cell HLA-I epitope), helix 4 with A125Y (uncharacterized protein, T-cell HLA-II epitope) or SFF (chloride transporter, B-cell epitope), and helix 6 with V4Z (surface antigen, T-cell HLA-I epitope) or S8E (uncharacterized protein, T-cell HLA-II epitope).

**Figure 2:**
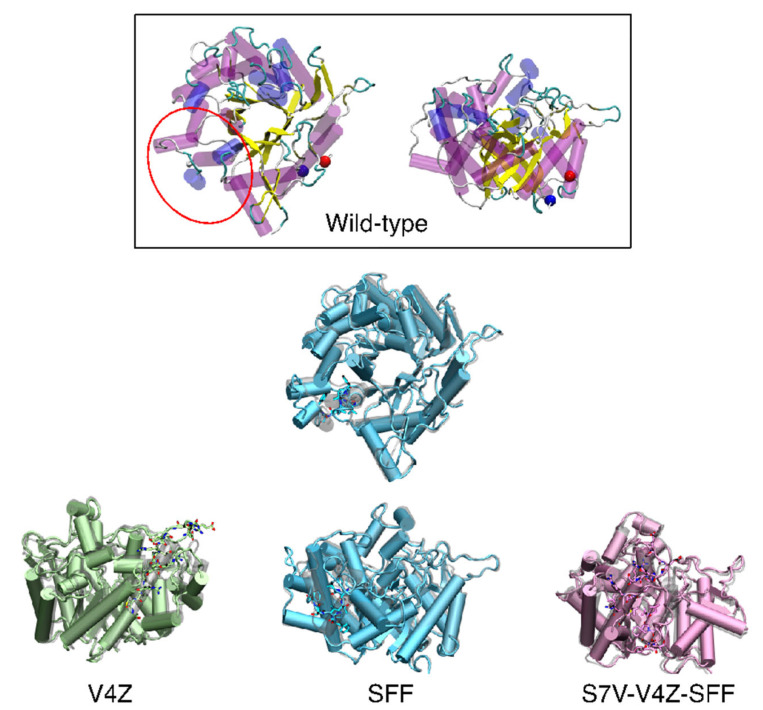
Superimposition between wild-type (grey) and the promising mutations, V4Z (green), SFF (light blue), and S7V-V4Z-SFF (pink). The mutation loop showed in licorice.

Structural modeling revealed that these substitutions produced various structural shifts, especially in the outer loop regions (Figure 2). The V4Z insertion largely retained the TIM-barrel structure, while SFF insertion showed slight deviations in inner loop regions. Triple epitope insertions, such as S7V-V4Z-SFF, induced more pronounced structural changes. These modifications are expected to influence the immunogenic properties of the proteins, potentially impacting their antigen–antibody binding capacity.

### 3.2. Serological Test

The primary indirect ELISA testing of positive and negative cat sera from Suwan et al. [19] revealed that a protein concentration of 0.5 µg/mL provided optimal differentiation between positive and negative samples, as evidenced by OD_655 nm_ values in Table S2. In contrast, concentrations of 0.75 and 1.5 µg/mL resulted in excessively high OD_655 nm_ readings, impairing the effective distinction between positive and negative samples. Among the 10 chimeric proteins at 0.5 µg/mL concentration, S7V-V4Z-SFF, SFF, and V4Z exhibited potential for detecting antibodies against *T. gondii* in cat sera.

The chimeric proteins S7V-V4Z-SFF, SFF, and V4Z, at a concentration of 0.5 µg/mL, were subsequently evaluated using 39 cat serum samples. ELISA-SFF displayed the highest OD_655 nm_ for positive (0.751) and negative (0.198) controls, followed by ELISA-V4Z (0.549 and 0.154) and ELISA-S7V-V4Z-SFF (0.523 and 0.116). The average intra-assay CV was 6.02%, 5.24%, and 5.49% for ELISA-S7V-V4Z-SFF, ELISA-SFF, and ELISA-V4Z, respectively, as presented in Table S3. CV < 10% is generally considered as high repeatability [45].

### 3.3. Evaluation of Indirect ELISA Assays

**Figure 3:**
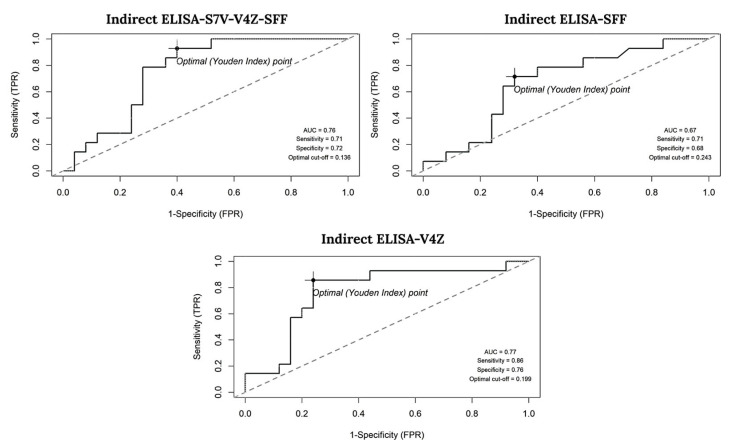
Receiver operating characteristic (ROC) curve illustrating the area under the curve (AUC) and the optimal cutoff point that achieves the highest sensitivity and specificity.

The performance of each indirect ELISA assay at various cutoff points was assessed using the ROC curve to determine the optimal cutoff, ensuring distinction between positive and negative samples while maintaining appropriate test sensitivity and specificity. The optimal cutoff was determined by analyzing the OD of 39 cat serum samples tested with ELISA-S7V-V4Z-SFF, ELISA-SFF, and ELISA-V4Z, compared to the IFAT results from Suwan et al. [19]. The ROC-based model identified optimal cutoff values of 0.136, 0.243, and 0.199 for ELISA-S7V-V4Z-SFF, ELISA-SFF, and ELISA-V4Z, respectively, as illustrated in Figure 3.

Sensitivity and Specificity: Among the assays, ELISA-V4Z achieved the highest sensitivity (86%, 95% CI: 57–98%) and specificity (76%, 95% CI: 55–91%). In contrast, ELISA-S7V-V4Z-SFF showed balanced performance, with sensitivity and specificity of 71% (95% CI: 42–92%) and 72% (95% CI: 51–88%), respectively. ELISA-SFF also exhibited moderate sensitivity (71%) but a lower specificity of 68% (95% CI: 39–79%).

Predictive Values: ELISA-V4Z demonstrated the highest positive predictive value (PPV) at 67% (95% CI: 41–87%) among the assays and achieved the strongest negative predictive value (NPV) of 90% (95% CI: 70–99%), as indicated in Table 1.

Prevalence Comparison: Among the three indirect ELISA assays, both indirect ELISA-SFF and ELISA-V4Z exhibited the highest prevalence at 46.1% (95% CI = 31.6–61.4%). Meanwhile, the indirect ELISA-S7V-V4Z-SFF demonstrated a slightly lower prevalence at 43.6% (95% CI = 29.3–59.0%). In contrast, the prevalence detected using IFAT was the lowest at 35.9% (95% CI = 22.7–51.6%). However, McNemar’s test indicated no statistically significant difference between IFAT and any of the indirect ELISA assays, nor among the indirect ELISA assays themselves (Table 2).

Analysis of Agreement: The concordance assessment between the indirect ELISA and IFAT methods demonstrated that ELISA-V4Z exhibited the highest agreement with IFAT (Kappa = 0.58, 95% CI: 0.32–0.84), indicating moderate concordance. This was followed by ELISA-S7V-V4Z-SFF, which showed a Kappa value of 0.41 (95% CI: 0.12–0.71), also indicating moderate concordance. In contrast, ELISA-SFF achieved a Kappa value of 0.37 (95% CI: 0.07–0.67), reflecting fair concordance. The PABAK values corresponded with the Kappa estimates across all three assessments, affirming consistency in agreement measures (Table 3).

## 4. Discussion

In this study, we engineered 10 chimeric multi-epitope proteins based on the TIM-barrel structure of Br2 *β*-glucosidase by substituting selected *α*-helices with B-cell and T-cell epitopes from *T. gondii*. These substitutions were confirmed by sequence alignment, and the resulting proteins were modeled to assess potential structural impacts. The multi-epitope proteins V4Z, S7V-V4Z-SFF, and SFF exhibited promising sensitivity and specificity in distinguishing between *T. gondii*-positive and -negative samples in ELISA assays. The V4Z revealed the highest sensitivity, specificity, positive predictive value, and negative predictive value due to its detectable antigenicity. Structural analysis revealed that these engineered proteins retained the TIM-barrel’s core stability, a *β*-sheet core encased in *α*-helices containing B- and T-cell epitopes, with targeted mutations enhancing epitope facet binds to other proteins, peptides, or substrates.

Serological methods, particularly ELISA, have long been used to diagnose toxoplasmosis in humans and animals, with significant advancements in antigen development over recent years. ELISAs are practical and cost-effective, making them suitable for routine *T. gondii* diagnosis. Commercially available ELISAs and in-house adaptations often utilize recombinant or chimeric antigens, which are increasingly favored due to their high specificity and sensitivity compared to a single antigen [13,15,16]. Advances in epitope mapping techniques such as immunoproteomics [46], protein microarrays [1], and two-dimensional gel electrophoresis [47] have expanded the potential for identifying and incorporating multiple antigenic regions within chimeric proteins. Computational approaches allow for the precise prediction of immunoreactive B- and T-cell epitopes facilitating the design of antigens with enhanced diagnostic properties [33]. However, the effectiveness of these in silico predictions requires rigorousness in vitro validation. Chimeric antigens, which combine multiple epitopes, have emerged as promising tools for *T. gondii* detection in various species, offering enhanced accuracy in livestock diagnosis [29,30,31].

The diagnostic performance of our chimeric proteins did not reach the high efficacy reported for tetravalent and trivalent chimeric proteins [30,31]. The moderate sensitivity and specificity achieved in this study suggest that further optimization, such as including additional immunodominant epitopes, could enhance their diagnostic potential. Variability in immune responses among different host species also underscores the need for tailored antigen designs, suggesting that further testing across multiple animal models could support species-specific ELISA optimization [29,30]. Moreover, the relatively small sample size may limit the statistical power and generalizability of the present findings; therefore, the diagnostic performance values should be interpreted with caution and regarded as preliminary until validated in larger and more diverse feline populations. Additionally, potential false-positive outcomes may result from cross-reactivity with antibodies against other pathogens, particularly those sharing similar epitopes [48,49], while false-negative results may occur due to immune suppression in the host, leading to weak or undetectable antibody responses [50]. These limitations could affect diagnostic accuracy and should be carefully considered in interpreting the results. Furthermore, the lack of confirmatory diagnostic methods, such as PCR, constrains the validation of ELISA results and contributes to uncertainty in interpreting serological data. Although intra-assay consistency was monitored, the reproducibility of the ELISA across different runs and personnel was not systematically assessed, potentially impacting its reliability under varied laboratory conditions. These limitations collectively highlight the importance of future investigations employing confirmatory diagnostics and standardized reproducibility evaluations to strengthen diagnostic confidence. Accordingly, we recommend that future studies incorporate broader pathogen panels and account for the health status of cat populations to enhance assay validation and refinement.

## 5. Conclusions

In this study, we successfully designed and evaluated a TIM-barrel-based multi-epitope protein by incorporating selected *T. gondii* B- and T-cell epitopes for serological diagnostics in cats. Our findings, particularly with the V4Z chimeric protein, demonstrate the potential of this approach to enhance diagnostic accuracy for *T. gondii* detection, exhibiting favorable sensitivity, specificity, and moderate agreement with the reference IFAT. While these initial results are encouraging and suggest that TIM-barrel-based multi-epitope proteins represent a valuable new strategy, further comprehensive studies are essential to validate these findings across diverse feline populations, confirm assay reproducibility, and fully assess its applicability for broader serological testing.

## Figures and Tables

**Table 1:** Sensitivity, specificity, positive predictive value (PPV) and negative predictive value (NPV) of each indirect ELISA when using IFAT as a reference test.

Indirect ELISA	Sensitivity		Specificity		PPV		NPV	
	%	95% CI	%	95% CI	%	95% CI	%	95% CI
S7V-V4Z-SFF	71	42–92	72	51–88	59	33–82	82	60–95
SFF	71	42–92	68	46–85	56	31–78	81	58–95
V4Z	86	57–98	76	55–91	67	41–87	90	67–99

**Table 2:** The *T. gondii* indirect ELISA results against 39 cat sera compared with IFAT and another ELISA assay.

Assay	Number of Positive Sample	Number of Negative Sample	Prevalence [95% CI]	McNemar’s χ2	*p*-Value
	ELISA-S7V-V4Z-SFF compared with IFAT				
ELISA-S7V-V4Z-SFF	17	22	43.6% [29.3–59.0]	1.17	0.28
IFAT	14	25	35.9% [22.7–51.6]		
	ELISA-SFF compared with IFAT				
ELISA-SFF	18	21	46.1% [31.6–61.4]	0.84	0.36
IFAT	14	25	35.9% [22.7–51.6]		
	ELISA-V4Z compared with IFAT				
ELISA-V4Z	18	21	46.1% [31.6–61.4]	0.84	0.36
IFAT	14	25	35.9% [22.7–51.6]		
	ELISA-S7V-V4Z-SFF compared with ELISA-SFF				
ELISA-S7V-V4Z-SFF	17	22	43.6% [29.3–59.0]	0.23	0.63
ELISA-SFF	18	21	46.1% [31.6–61.4]		
	ELISA-S7V-V4Z-SFF compared with ELISA-V4Z				
ELISA-S7V-V4Z-SFF	17	22	43.6% [29.3–59.0]	0.23	0.63
ELISA-V4Z	18	21	46.1% [31.6–61.4]		
	ELISA-VH5-SFF compared with ELISA-V4Z				
ELISA-SFF	18	21	46.1% [31.6–61.4]	0.10	0.75
ELISA-V4Z	18	21	46.1% [31.6–61.4]		

**Table 3:** The analysis of agreement of the indirect ELISA using IFAT as a reference test.

	Kappa Value [95% CI]	PABAK Value * [95% CI]	Strength of Agreement
ELISA-S7V-V4Z-SFF vs. IFAT	0.41 [0.12–0.71]	0.43 [0.10–0.70]	Moderate agreement
ELIS-SFF vs. IFAT	0.37 [0.07–0.67]	0.38 [0.05–0.66]	Fair agreement
ELIS-V4Z vs. IFAT	0.58 [0.32–0.84]	0.59 [0.27–0.81]	Moderate agreement

* The prevalence-adjusted bias-adjusted Kappa.

## Data Availability

The original contributions presented in this study are included in the article and Supplementary Materials. Further inquiries can be directed to the corresponding author.

## Supplementary Materials

The following supporting information can be downloaded at: https://www.mdpi.com/article/10.3390/ani15131893/s1, Table S1: The set of deletions and insertions primer used in this study; Table S2: The absorbance at 655 nm of in cat serum for *T. gondii* detection via indirect ELISA; Table S3: The absorbance at 655 nm of *T. gondii* indirect ELISA from each protein; Table S4: The percentage of 11 sequences alignments.
