# Supplementary material for: The Development of TIM-Barrel Based Multi-Epitope Protein for Toxoplasma gondii Serological Detection in Cats
Source: Animals (Basel). 2025 Jun 26;15(13):1893. doi: 10.3390/ani15131893 (PMC12249241; doi:10.3390/ani15131893)
Supplement: Supplementary file 1 [file animals-15-01893-s001.zip › animals-3672956-supplementary.pdf]

**Supplementary data: The development of TIM-barrel based multi-epitope protein for *Toxoplasma gondii* serological detection in cats**

Preeyanuch Thongpoo[a], Jiravich Methawiroon[b], Bandid Mangkit[b], Rucksak Rucksaken[c], Metita Sussadee[c], Warin Rangubpit[b,d,e], Sathaporn Jittapalapong[b,d,f], and Eukote Suwan[b,d,*]

**Table S1** The set of deletions and insertions primer used in this study.

| Name | Sequence (5' – 3') | Description |
|---|---|---|
| Deletion primer | | |
| H3del_F | GGGAAAGAAGTATCTTACTTTATC | Helix 3 Br2 deletion |
| H3del_R | ATATTCAGCAAACCAATTAG | |
| H4del_F | CTTGCAGTGAAAGAGTTG | Helix 4 Br2 deletion |
| H4del_R | CTTTGGTAATTGATATCCTGG | |
| H6del_F | AAATGGGTAACCTACATAAAC | Helix 6 Br2 deletion |
| H6del_R | GAAAACAAATTCGTTGCTATTAC | |
| Insertion primer | | |
| S7V_F | GTGACCTTTCTGGGGAAAGAAGTATCTTACTTTATC | Guanylate cyclase, T-cell HLA-I epitope, Helix 3 insertion |
| S7V_R | GTTTTCATGCAGATATTCAGCAAACCAATTAG | |
| A125Y_F | CGGCGGCGCTGGCGGCGGCGCGCCTTGCAGTGAAAGAGTTG | Uncharacterized protein, T-cell HLA-II epitope, Helix 4 insertion |
| A125Y_R | CGCTCAGGCGCAGGCGATCTTCCTTTGGTAATTGATATCCTGG | |
| SFF_F | GCGATAGCCGCGATGTGCGCCTGTGCACCCTTGCAGTGAAAGAGTTG | Chloride transporter, B-cell epitope, Helix 4 insertion |
| SFF_R | GGCTATCGCCGCGGCCTTCGCCTTCCGCCTTTGGTAATTGATATCCTG G | |
| S8E_F | ATCTGTTTCGTATTCGTTATAGCAAATGGGTAACCTACATAAAC | Uncharacterized protein, T-cell HLA-II epitope, Helix 6 insertion |
| S8E_R | GAAAATACAGAAAAATCGGAATGAAAACAAATTCGTTGCTATTAC | |
| V4Z_F | GTTTGTGAGCTTTAAATGGGTAACCTACATAAAC | Surface antigen (SAG), T-cell HLA-I epitope, Helix 6 insertion |
| V4Z_R | GGGGTAAACACCACGAAAACAAATTCGTTGCTATTAC | |

**Table S2** The $OD_{655\ nm}$ values of each chimeric protein concentration tested with positive and negative cat sera using indirect ELISA for *Toxoplasma gondii* infection detection.

| Sample/Protein | Br2 | S8E | S7V-V4Z | S7V-V4Z-SFF* | S7V | SFF* | S7V-S8E-SFF | V4Z* | S7V-S8E | S7V-S8E-A125Y | A125Y |
|---|---|---|---|---|---|---|---|---|---|---|---|
| **Protein concentration of 1.5 µg/mL** | | | | | | | | | | | |
| Positive 1 | 1.085 | 1.955 | 1.803 | 1.214 | 1.664 | 1.422 | 1.740 | 2.260 | 0.187 | 1.782 | 0.748 |
| Positive 2 | 1.165 | 2.099 | 1.900 | 1.487 | 1.823 | 1.436 | 1.870 | 2.263 | 2.299 | 2.030 | 0.862 |
| Positive 3 | 0.913 | 1.961 | 1.820 | 1.268 | 1.805 | 1.155 | 1.619 | 2.184 | 2.132 | 1.693 | 0.377 |
| Negative 1 | 0.481 | 1.365 | 1.075 | 0.650 | 1.044 | 0.736 | 1.262 | 1.081 | 1.404 | 1.174 | 0.294 |
| Negative 2 | 1.173 | 1.979 | 1.774 | 1.486 | 1.539 | 1.122 | 1.639 | 1.837 | 1.994 | 1.836 | 0.592 |
| Negative 3 | 0.799 | 2.026 | 1.799 | 1.245 | 1.641 | 1.139 | 1.392 | 1.830 | 1.924 | 1.349 | 0.399 |
| **Protein concentration of 0.75 µg/mL** | | | | | | | | | | | |
| Positive 1 | 0.433 | 0.805 | 0.793 | 0.473 | 0.919 | 0.715 | 1.236 | 0.532 | 0.630 | 0.725 | 0.155 |
| Positive 2 | 0.650 | 0.989 | 0.980 | 0.656 | 1.087 | 0.880 | 1.154 | 0.705 | 0.792 | 0.796 | 0.348 |
| Positive 3 | 0.298 | 0.559 | 0.620 | 0.380 | 0.861 | 0.584 | 0.817 | 0.344 | 0.475 | 0.456 | 0.104 |
| Negative 1 | 0.101 | 0.731 | 0.820 | 0.491 | 0.859 | 0.643 | 0.783 | 0.122 | 0.219 | 0.276 | 0.085 |
| Negative 2 | 0.474 | 0.681 | 0.770 | 0.441 | 0.809 | 0.593 | 0.733 | 0.268 | 0.739 | 0.703 | 0.073 |
| Negative 3 | 0.284 | 0.624 | 0.643 | 0.345 | 0.660 | 0.674 | 0.705 | 0.325 | 0.446 | 0.446 | 0.080 |
| **Protein concentration of 0.50 µg/mL** | | | | | | | | | | | |
| Positive 1 | 0.097 | 0.340 | 0.180 | 0.225 | 0.193 | 0.351 | 0.541 | 0.294 | 0.427 | 0.342 | 0.101 |
| Positive 2 | 0.099 | 0.516 | 0.354 | 0.305 | 0.510 | 0.407 | 0.541 | 0.590 | 0.638 | 0.641 | 0.222 |
| Positive 3 | 0.099 | 0.190 | 0.130 | 0.187 | 0.088 | 0.374 | 0.478 | 0.301 | 0.451 | 0.371 | 0.104 |
| Negative 1 | 0.028 | 0.100 | 0.053 | 0.040 | 0.041 | 0.074 | 0.166 | 0.113 | 0.177 | 0.131 | 0.093 |
| Negative 2 | 0.060 | 0.185 | 0.091 | 0.128 | 0.069 | 0.121 | 0.380 | 0.147 | 0.486 | 0.502 | 0.060 |
| Negative 3 | 0.061 | 0.157 | 0.083 | 0.125 | 0.099 | 0.091 | 0.444 | 0.131 | 0.372 | 0.332 | 0.048 |

* indicated the suitable chimeric proteins for further study.

**Table S3** The $OD_{655\ nm}$ values of *T. gondii* indirect ELISA from chimeric proteins S7V-V4Z-SFF, SFF, and V4Z, at a concentration of 0.5 µg/mL, using 39 cat serum samples.

| Sample | Status | Result 1 | Result 2 | Mean | Standard Deviation | % Coefficient of Variation (CV) |
|---|---|---|---|---|---|---|
| **Indirect ELISA-*S7V-V4Z-SFF*** | | | | | | |
| F633 | Positive Control 1 | 0.551 | 0.537 | 0.544 | 0.010 | 1.82 |
| F634 | Positive Control 2 | 0.498 | 0.505 | 0.502 | 0.005 | 0.99 |
| Mean | | 0.525 | 0.521 | 0.523 | | |
| F643 | Negative Control 1 | 0.121 | 0.127 | 0.124 | 0.004 | 3.42 |
| F644 | Negative Control 2 | 0.118 | 0.126 | 0.122 | 0.006 | 4.64 |
| F638 | Negative Control 3 | 0.056 | 0.058 | 0.057 | 0.001 | 2.48 |
| F642 | Negative Control 4 | 0.200 | 0.123 | 0.162 | 0.054 | 33.71 |
| Mean | | 0.124 | 0.109 | 0.116 | | |
| CDV-003 | True Negative | 0.222 | 0.195 | 0.209 | 0.019 | 9.16 |
| CDV-004 | True Negative | 0.278 | 0.251 | 0.265 | 0.019 | 7.22 |
| CDV-005 | True Negative | 0.121 | 0.130 | 0.126 | 0.006 | 5.07 |
| CDV-006 | True Negative | 0.126 | 0.128 | 0.127 | 0.001 | 1.11 |
| STB-010 | True Negative | 0.086 | 0.078 | 0.082 | 0.006 | 6.90 |
| STB-011 | True Negative | 0.330 | 0.355 | 0.343 | 0.018 | 5.16 |
| STB-013 | True Negative | 0.095 | 0.107 | 0.101 | 0.008 | 8.40 |
| STB-014 | True Negative | 0.170 | 0.142 | 0.156 | 0.020 | 12.69 |
| N-002 | True Negative | 0.094 | 0.092 | 0.093 | 0.001 | 1.52 |
| N-004 | True Negative | 0.495 | 0.458 | 0.477 | 0.026 | 5.49 |

| | | | | | | |
|---|---|---|---|---|---|---|
| J-001 | True Negative | 0.101 | 0.100 | 0.101 | 0.001 | 0.70 |
| BB-002 | True Negative | 0.255 | 0.250 | 0.253 | 0.004 | 1.40 |
| PS-003 | True Negative | 0.083 | 0.085 | 0.084 | 0.001 | 1.68 |
| S-006 | True Negative | 0.077 | 0.084 | 0.081 | 0.005 | 6.15 |
| S-007 | True Negative | 0.129 | 0.123 | 0.126 | 0.004 | 3.37 |
| L-001 | True Negative | 0.137 | 0.142 | 0.140 | 0.004 | 2.53 |
| CYG-005 | True Negative | 0.100 | 0.094 | 0.097 | 0.004 | 4.37 |
| Sam-005 | True Negative | 0.072 | 0.071 | 0.072 | 0.001 | 0.99 |
| YS-003 | True Negative | 0.076 | 0.076 | 0.076 | 0.000 | 0.00 |
| YS-004 | True Negative | 0.050 | 0.054 | 0.052 | 0.003 | 5.44 |
| CDV-037 | True Positive | 0.266 | 0.272 | 0.269 | 0.004 | 1.58 |
| CDV-048 | True Positive | 0.411 | 0.385 | 0.398 | 0.018 | 4.62 |
| CDV-081 | True Positive | 0.253 | 0.239 | 0.246 | 0.010 | 4.02 |
| CDV-084 | True Positive | 0.291 | 0.272 | 0.282 | 0.013 | 4.77 |
| STB-005 | True Positive | 0.339 | 0.353 | 0.346 | 0.010 | 2.86 |
| STB-012 | True Positive | 0.197 | 0.200 | 0.199 | 0.002 | 1.07 |
| STB-016 | True Positive | 0.208 | 0.217 | 0.213 | 0.006 | 2.99 |
| PS-013 | True Positive | 0.176 | 0.157 | 0.167 | 0.013 | 8.07 |
| S-002 | True Positive | 0.145 | 0.128 | 0.137 | 0.012 | 8.81 |
| S-004 | True Positive | 0.157 | 0.152 | 0.155 | 0.004 | 2.29 |
| S-005 | True Positive | 0.190 | 0.186 | 0.188 | 0.003 | 1.50 |
| S-019 | True Positive | 0.119 | 0.119 | 0.119 | 0.000 | 0.00 |

| | | | | | | |
|---|---|---|---|---|---|---|
| L-005 | True Positive | 0.247 | 0.221 | 0.234 | 0.018 | 7.86 |
| CDV-083 | False Negative | 0.205 | 0.173 | 0.189 | 0.023 | 11.97 |
| CDV-049 | False Positive | 0.177 | 0.153 | 0.165 | 0.017 | 10.29 |
| S-003 | False Positive | 0.312 | 0.241 | 0.277 | 0.050 | 18.16 |
| S-011 | False Positive | 0.297 | 0.240 | 0.269 | 0.040 | 15.01 |
| L-009 | False Positive | 0.104 | 0.074 | 0.089 | 0.021 | 23.84 |
| YS-002 | False Positive | 0.094 | 0.088 | 0.091 | 0.004 | 4.66 |
| **Mean** | | **0.196** | **0.186** | **0.191** | **0.011** | **6.02** |
| **Indirect ELISA-*SFF*** | | | | | | |
| F633 | Positive Control 1 | 0.790 | 0.791 | 0.791 | 0.001 | 0.09 |
| F634 | Positive Control 2 | 0.723 | 0.698 | 0.711 | 0.018 | 2.49 |
| Mean | | 0.757 | 0.745 | 0.751 | | |
| F643 | Negative Control 1 | 0.170 | 0.166 | 0.168 | 0.003 | 1.68 |
| F644 | Negative Control 2 | 0.253 | 0.232 | 0.243 | 0.015 | 6.12 |
| F638 | Negative Control 3 | 0.174 | 0.164 | 0.169 | 0.007 | 4.18 |
| F642 | Negative Control 4 | 0.206 | 0.222 | 0.214 | 0.011 | 5.29 |
| Mean | | 0.201 | 0.196 | 0.198 | | |
| CDV-003 | True Negative | 0.388 | 0.404 | 0.396 | 0.011 | 2.86 |
| CDV-004 | True Negative | 0.449 | 0.446 | 0.448 | 0.002 | 0.47 |
| CDV-005 | True Negative | 0.168 | 0.173 | 0.171 | 0.004 | 2.07 |
| CDV-006 | True Negative | 0.120 | 0.113 | 0.117 | 0.005 | 4.25 |
| STB-010 | True Negative | 0.170 | 0.168 | 0.169 | 0.001 | 0.84 |

| | | | | | | |
|---|---|---|---|---|---|---|
| STB-011 | True Negative | 0.571 | 0.513 | 0.542 | 0.041 | 7.57 |
| STB-013 | True Negative | 0.131 | 0.128 | 0.130 | 0.002 | 1.64 |
| STB-014 | True Negative | 0.201 | 0.178 | 0.190 | 0.016 | 8.58 |
| N-002 | True Negative | 0.104 | 0.101 | 0.103 | 0.002 | 2.07 |
| N-004 | True Negative | 0.621 | 0.566 | 0.594 | 0.039 | 6.55 |
| J-001 | True Negative | 0.159 | 0.149 | 0.154 | 0.007 | 4.59 |
| BB-002 | True Negative | 0.360 | 0.356 | 0.358 | 0.003 | 0.79 |
| PS-003 | True Negative | 0.123 | 0.119 | 0.121 | 0.003 | 2.34 |
| S-006 | True Negative | 0.109 | 0.096 | 0.103 | 0.009 | 8.97 |
| S-007 | True Negative | 0.143 | 0.139 | 0.141 | 0.003 | 2.01 |
| L-001 | True Negative | 0.297 | 0.299 | 0.298 | 0.001 | 0.47 |
| CYG-005 | True Negative | 0.173 | 0.147 | 0.160 | 0.018 | 11.49 |
| Sam-005 | True Negative | 0.088 | 0.086 | 0.087 | 0.001 | 1.63 |
| YS-003 | True Negative | 0.069 | 0.080 | 0.075 | 0.008 | 10.44 |
| YS-004 | True Negative | 0.052 | 0.053 | 0.053 | 0.001 | 1.35 |
| CDV-037 | True Positive | 0.382 | 0.376 | 0.379 | 0.004 | 1.12 |
| CDV-048 | True Positive | 0.522 | 0.492 | 0.507 | 0.021 | 4.18 |
| CDV-081 | True Positive | 0.361 | 0.351 | 0.356 | 0.007 | 1.99 |
| CDV-084 | True Positive | 0.407 | 0.406 | 0.407 | 0.001 | 0.17 |
| STB-005 | True Positive | 0.378 | 0.326 | 0.352 | 0.037 | 10.45 |
| STB-012 | True Positive | 0.256 | 0.285 | 0.271 | 0.021 | 7.58 |
| STB-016 | True Positive | 0.363 | 0.396 | 0.380 | 0.023 | 6.15 |

| | | | | | | |
|---|---|---|---|---|---|---|
| PS-013 | True Positive | 0.096 | 0.094 | 0.095 | 0.001 | 1.49 |
| S-002 | True Positive | 0.170 | 0.174 | 0.172 | 0.003 | 1.64 |
| S-004 | True Positive | 0.117 | 0.125 | 0.121 | 0.006 | 4.68 |
| S-005 | True Positive | 1.243 | 1.255 | 1.249 | 0.008 | 0.68 |
| S-019 | True Positive | 0.139 | 0.156 | 0.148 | 0.012 | 8.15 |
| L-005 | True Positive | 0.356 | 0.326 | 0.341 | 0.021 | 6.22 |
| CDV-083 | False Negative | 0.416 | 0.355 | 0.386 | 0.043 | 11.19 |
| CDV-049 | False Positive | 0.272 | 0.238 | 0.255 | 0.024 | 9.43 |
| S-003 | False Positive | 0.415 | 0.389 | 0.402 | 0.018 | 4.57 |
| S-011 | False Positive | 0.458 | 0.363 | 0.411 | 0.067 | 16.36 |
| L-009 | False Positive | 0.099 | 0.070 | 0.085 | 0.021 | 24.27 |
| YS-002 | False Positive | 0.151 | 0.123 | 0.137 | 0.020 | 14.45 |
| **Mean** | | **0.298** | **0.286** | **0.292** | **0.013** | **5.24** |
| **Indirect ELISA-*V4Z*** | | | | | | |
| F633 | Positive Control 1 | 0.477 | 0.472 | 0.475 | 0.004 | 0.75 |
| F634 | Positive Control 2 | 0.634 | 0.614 | 0.624 | 0.014 | 2.27 |
| Mean | | 0.556 | 0.543 | 0.549 | | |
| F643 | Negative Control 1 | 0.118 | 0.123 | 0.121 | 0.004 | 2.93 |
| F644 | Negative Control 2 | 0.196 | 0.182 | 0.189 | 0.010 | 5.24 |
| F638 | Negative Control 3 | 0.139 | 0.139 | 0.139 | 0.000 | 0.00 |
| F642 | Negative Control 4 | 0.165 | 0.167 | 0.166 | 0.001 | 0.85 |
| Mean | | 0.155 | 0.153 | 0.154 | | |

| | | | | | | |
|---|---|---|---|---|---|---|
| CDV-003 | True Negative | 0.231 | 0.198 | 0.215 | 0.023 | 10.88 |
| CDV-004 | True Negative | 0.306 | 0.339 | 0.323 | 0.023 | 7.24 |
| CDV-005 | True Negative | 0.235 | 0.194 | 0.215 | 0.029 | 13.52 |
| CDV-006 | True Negative | 0.190 | 0.172 | 0.181 | 0.013 | 7.03 |
| STB-010 | True Negative | 0.100 | 0.086 | 0.093 | 0.010 | 10.64 |
| STB-011 | True Negative | 0.388 | 0.430 | 0.409 | 0.030 | 7.26 |
| STB-013 | True Negative | 0.152 | 0.148 | 0.150 | 0.003 | 1.89 |
| STB-014 | True Negative | 0.195 | 0.192 | 0.194 | 0.002 | 1.10 |
| N-002 | True Negative | 0.110 | 0.109 | 0.110 | 0.001 | 0.65 |
| N-004 | True Negative | 0.434 | 0.458 | 0.446 | 0.017 | 3.81 |
| J-001 | True Negative | 0.157 | 0.137 | 0.147 | 0.014 | 9.62 |
| BB-002 | True Negative | 0.285 | 0.263 | 0.274 | 0.016 | 5.68 |
| PS-003 | True Negative | 0.092 | 0.114 | 0.103 | 0.016 | 15.10 |
| S-006 | True Negative | 0.178 | 0.134 | 0.156 | 0.031 | 19.94 |
| S-007 | True Negative | 0.151 | 0.136 | 0.144 | 0.011 | 7.39 |
| L-001 | True Negative | 0.221 | 0.203 | 0.212 | 0.013 | 6.00 |
| CYG-005 | True Negative | 0.116 | 0.115 | 0.116 | 0.001 | 0.61 |
| Sam-005 | True Negative | 0.104 | 0.124 | 0.114 | 0.014 | 12.41 |
| YS-003 | True Negative | 0.138 | 0.117 | 0.128 | 0.015 | 11.65 |
| YS-004 | True Negative | 0.080 | 0.078 | 0.079 | 0.001 | 1.79 |
| CDV-037 | True Positive | 0.368 | 0.381 | 0.375 | 0.009 | 2.45 |
| CDV-048 | True Positive | 0.566 | 0.546 | 0.556 | 0.014 | 2.54 |

| | | | | | | |
|---|---|---|---|---|---|---|
| CDV-081 | True Positive | 0.334 | 0.342 | 0.338 | 0.006 | 1.67 |
| CDV-084 | True Positive | 0.379 | 0.357 | 0.368 | 0.016 | 4.23 |
| STB-005 | True Positive | 0.343 | 0.378 | 0.361 | 0.025 | 6.87 |
| STB-012 | True Positive | 0.236 | 0.248 | 0.242 | 0.008 | 3.51 |
| STB-016 | True Positive | 0.387 | 0.434 | 0.411 | 0.033 | 8.10 |
| PS-013 | True Positive | 0.101 | 0.097 | 0.099 | 0.003 | 2.86 |
| S-002 | True Positive | 0.231 | 0.227 | 0.229 | 0.003 | 1.24 |
| S-004 | True Positive | 0.189 | 0.176 | 0.183 | 0.009 | 5.04 |
| S-005 | True Positive | 1.237 | 1.364 | 1.301 | 0.090 | 6.91 |
| S-019 | True Positive | 0.265 | 0.270 | 0.268 | 0.004 | 1.32 |
| L-005 | True Positive | 0.313 | 0.364 | 0.339 | 0.036 | 10.65 |
| CDV-083 | False Negative | 0.289 | 0.309 | 0.299 | 0.014 | 4.73 |
| CDV-049 | False Positive | 0.214 | 0.235 | 0.225 | 0.015 | 6.61 |
| S-003 | False Positive | 0.443 | 0.458 | 0.451 | 0.011 | 2.35 |
| S-011 | False Positive | 0.455 | 0.443 | 0.449 | 0.008 | 1.89 |
| L-009 | False Positive | 0.152 | 0.138 | 0.145 | 0.010 | 6.83 |
| YS-002 | False Positive | 0.134 | 0.132 | 0.133 | 0.001 | 1.06 |
| **Mean** | | **0.272** | **0.274** | **0.273** | **0.014** | **5.49** |

**Table S4:** The percentage of 11 sequences alignments

| | WT | S7V | S8E | V4Z | SFF | A125Y | S7V-V4Z | S7V-S8E | S7V-S8E-SFF | S7V-S8E-A125Y | S7V-V4Z-SFF |
|---|---|---|---|---|---|---|---|---|---|---|---|
| WT | 100.00 | 98.43 | 97.30 | 97.08 | 96.44 | 97.30 | 95.51 | 95.73 | 92.22 | 93.03 | 92.00 |
| S7V | | 100.00 | 95.73 | 95.51 | 94.89 | 95.73 | 97.08 | 97.30 | 93.78 | 94.61 | 93.56 |
| S8E | | | 100.00 | 97.08 | 93.78 | 94.61 | 95.51 | 98.43 | 94.89 | 95.73 | 92.00 |
| V4Z | | | | 100 | 93.56 | 94.38 | 98.41 | 95.51 | 92.00 | 92.81 | 94.82 |
| SFF | | | | | 100.00 | 96.44 | 92.00 | 92.22 | 95.77 | 92.20 | 95.55 |
| A125Y | | | | | | 100.00 | 92.81 | 93.03 | 91.98 | 95.73 | 91.76 |
| S7V-V4Z | | | | | | | 100.00 | 97.08 | 93.56 | 94.38 | 96.40 |
| S7V-S8E | | | | | | | | 100.00 | 96.44 | 97.30 | 93.56 |
| S7V-S8E-SFF | | | | | | | | | 100.00 | 96.44 | 97.10 |
| S7V-S8E-A125Y | | | | | | | | | | 100.00 | 93.32 |
| S7V-V4Z-SFF | | | | | | | | | | | 100.00 |
